# Comparative Transcriptome Profiling Reveals Defense-Related Genes Against *Ralstonia solanacearum* Infection in Tobacco

**DOI:** 10.3389/fpls.2021.767882

**Published:** 2021-12-14

**Authors:** Xiaoying Pan, Junbiao Chen, Aiguo Yang, Qinghua Yuan, Weicai Zhao, Tingyu Xu, Bowen Chen, Min Ren, Ruimei Geng, Zhaohui Zong, Zhuwen Ma, Zhenrui Huang, Zhenchen Zhang

**Affiliations:** ^1^Guangdong Provincial Engineering & Technology Research Center for Tobacco Breeding and Comprehensive Utilization, Guangdong Key Laboratory for Crops Genetic Improvement, Crops Research Institute, Guangdong Academy of Agricultural Sciences (GAAS), Guangzhou, China; ^2^Key Laboratory of Tobacco Improvement and Biotechnology, Tobacco Research Institute of Chinese Academy of Agricultural Sciences, Qingdao, China; ^3^Nanxiong Tobacco Science Institute of Guangdong, Nanxiong, China

**Keywords:** bacterial wilt, *Ralstonia solanacearum*, RNA sequencing, cell wall, hormone, tobacco

## Abstract

Bacterial wilt (BW) caused by *Ralstonia solanacearum* (*R. solanacearum*), is a vascular disease affecting diverse solanaceous crops and causing tremendous damage to crop production. However, our knowledge of the mechanism underlying its resistance or susceptibility is very limited. In this study, we characterized the physiological differences and compared the defense-related transcriptomes of two tobacco varieties, 4411-3 (highly resistant, HR) and K326 (moderately resistant, MR), after *R. solanacearum* infection at 0, 10, and 17 days after inoculation (dpi). A total of 3967 differentially expressed genes (DEGs) were identified between the HR and MR genotypes under mock condition at three time points, including1395 up-regulated genes in the HR genotype and 2640 up-regulated genes in the MR genotype. Also, 6,233 and 21,541 DEGs were induced in the HR and MR genotypes after *R. solanacearum* infection, respectively. Furthermore, GO and KEGG analyses revealed that DEGs in the HR genotype were related to the cell wall, starch and sucrose metabolism, glutathione metabolism, ABC transporters, endocytosis, glycerolipid metabolism, and glycerophospholipid metabolism. The defense-related genes generally showed genotype-specific regulation and expression differences after *R. solanacearum* infection. In addition, genes related to auxin and ABA were dramatically up-regulated in the HR genotype. The contents of auxin and ABA in the MR genotype were significantly higher than those in the HR genotype after *R. solanacearum* infection, providing insight into the defense mechanisms of tobacco. Altogether, these results clarify the physiological and transcriptional regulation of *R. solanacearum* resistance infection in tobacco, and improve our understanding of the molecular mechanism underlying the plant-pathogen interaction.

## Introduction

Bacterial wilt (BW) is one of the most prevalent plant diseases, affecting hundreds of species, including agronomically important crops, such as tomato, chili pepper, sweet pepper, potato, eggplant, and tobacco belonging to the solanaceae family. The disease also affects non-solanaceous crops, like bananas, beans, and ornamental plants (Buddenhagen, 1986). BW is distributed worldwide in tropical and subtropical countries and is caused by the soilborne bacterial pathogen *R. solanacearum* (Hayward, 1991; Elphinstone et al., 2005). The mechanisms underlying natural resistance to *R. solanacearum* are related to the suppression of the growth and movement of the pathogen within the vascular system of their host (Xue et al., 2020). Histological analyses have revealed strengthened parenchyma cell walls and pit membranes in the xylem tissues and pathogen localization in the primary xylem tissues in the stems of a resistant tomato cultivar (Nakaho et al., 2000). In addition, the roles of cell wall proteins in defense against *R. solanacearum* in tomato have been discussed extensively (Wydra and Beri, 2006; Diogo and Wydra, 2007; Dahal et al., 2010; Schacht et al., 2011). Moreover, several recent studies have identified genes related to plant defense against *R. solanacearum*, including *StMKK1* in potato (Chen et al., 2021), *SlNAP1* and a bacterial effector protein RipAK in tomato (Wang et al., 2020; Wang Y. et al., 2021), and *CaNAC2c* in pepper (Cai et al., 2021).

Transcriptional profiling and analysis of gene function related to the host response to *R. solanacearum* are limited. Godiard et al. (1991) isolated cDNA clones corresponding to mRNAs that accumulate during the early phase of the hypersensitive response in suspension cultured tobacco cells challenged with a non-pathogenic strain of *R. solanacearum* (Godiard et al., 1991). Microarray analysis showed that R-response genes were related to xyloglucan biosynthesis and cell wall organization, while S-response genes were involved in response to stress and cell death in pepper (Hwang et al., 2011). A root transcriptome provided insight into the dynamic crosstalk between peanut and *R. solanacearum* (Chen et al., 2014). Several recent studies have evaluated root transcriptional responses during *R. solanacearum* infection in different plant species, including *Arabidopsis* (Zhao et al., 2019), tomato (French et al., 2018), and the wild potato *Solanum commersonii* (Zuluaga et al., 2015), revealing that auxin and ABA contribute to the infectivity of *R. solanacearum* and its ability to manipulate plant development.

Polygenic resistance patterns have been found in *Solanaceae* species, while monogenic inheritance was reported in *Arabidopsis thaliana* (Deslandes et al., 2002). The genetic basis of resistance to BW is similar in pepper and tomato. In tomato, several major QTL, particularly on chromosomes 4, 6, 8, and 12, are involved in the control of strain-specific resistance (Thoquet et al., 1996a,b; Wang et al., 2000; Carmeille et al., 2006). A quantitative character governed by oligogenic inheritance has also been reported to facilitate partial resistance to *R. solanacearum* in *Capsicum annuum* (Lafortune et al., 2005; Tran and Kim, 2010). Different modes of gene action among pathogen isolates with different levels of virulence have been reported in pepper, indicating the complex inheritance of BW resistance (Tran and Kim, 2010).

Several recent reports have provided a preliminary understanding of the molecular mechanism underlying the plant response to *R. solanacearum* by virus-induced gene silencing in tobacco. The suppression of DS1 (Disease suppression 1) function or *DS1* expression could rapidly activate plant defenses to achieve effective resistance against *R. solanacearum* in *Nicotiana benthamiana* (Nakano et al., 2013). NbPDKs played a crucial role in the regulation of hypersensitive cell death via plant hormone signaling and oxidative burst in the *NbPDK*-silenced plants challenged with *R. solanacearum* (Kiba et al., 2020). The *R. solanacearum* effector RipI induces a host defense reaction by interacting with the bHLH93 transcription factor in tobacco (Zhuo et al., 2020). The silencing of TOM20 (a marker of oxidative phosphorylation), PP1 (a protein phosphatase related to plant immune regulation), and HBP2 (a heme-binding protein related to the antioxidant pathway) in tobacco significantly altered resistance to *R. solanacearum* (Lee et al., 2012; Urvalek et al., 2015; Liu et al., 2019; Wang B. S. et al., 2021).

The mechanism underlying plant resistance to *R. solanacearum* is not clear. Moreover, BW is difficult to control due to its aggressiveness and the lack of resistant tobacco varieties in China. Tobacco cultivar GDSY-1, a Chinese domestic sun-cured tobacco variety, exhibits higher resistance to BW caused by *R. solanacearum* than other common tobacco cultivars (e.g., K326) carrying polygenic resistance derived from T.I.448A (Zhang et al., 2017). In this study, we measured physiological indexes in resistant (4411-3, carrying monogenic resistance derived from GDSY-1, referred to as HR) and moderately resistant (K326, referred to as MR) tobacco varieties. Further, we compared the defense transcriptomes and identified enriched GO and KEGG pathways, and hub genes associated with the tobacco response to *R. solanacearum* infection. Our results provide a valuable resource for understanding the interactions between tobacco and *R. solanacearum*.

## Materials and Methods

### Plant Culture and Growth Conditions

*Nicotiana tabacum* L. cv. 4411-3 (carrying monogenic resistance derived from GDSY-1) and *N. tabacum* L. cv. K326 were used. To eliminate the effect of different genetic backgrounds between K326 and GDSY-1, we used K326 as the female parent and crossed it with the male parent GDSY-1. The F_1_ plants were backcrossed to K326 (recurrent parent), and descendants were backcrossed to K326 four times to generate BC_4_F_5_ (4411-3), carrying monogenic resistance derived from GDSY-1, which was validated by BSA experiments. The final product, 4411-3 (K326-like type) was used for subsequent experiments.

Seeds were coated and germinated in a mixture of peat culture substrate, carbonized chaff, and perlite (3:2:1, V/V/V). The seedlings were grown in a naturally illuminated glasshouse for two months.

### Treatments and Sampling

A moderately aggressive defoliating strain of *R. solanacearum*, B-2 (Xie et al., 2014), was used for the disease assay. Five-leaf-stage tobacco plants were infected with 300 mL *R*. *solanacearum* cell suspension (3 × 10^7^ cell per mL) by irrigating roots in one pot. The roots of the control plants were irrigated with 300 mL distilled water. Tobacco seedlings of each variety were grown in five different pots, each containing 72 seedlings. Subsequently, the whole stem tissue of every plant was harvested. Tobacco stems from five individual seedlings taken from one pot were considered a biological replicate. Therefore, five biological replicates of each variety were harvested from five different pots at every time point. Three RNA samples from three biological replicates were used for RNA sequencing. Tobacco stems from five individual seedlings were taken from the inoculated and mock-inoculated plants at 0, 10, and 17 days post-inoculation (dpi). The samples were immediately frozen in liquid nitrogen and stored at −80°C for RNA exaction. Samples of five biological replicates were prepared.

The disease index was determined by GB/T 23224-2008 as described previously (Liu et al., 2017). The stems were weighed and ground using a mortar and pestle (with the addition of 10 mL sterile water) to measure their bacterial content. One mL supernatant was diluted five times after standing the ground samples 5 min. Next, 100 μL suspension was evenly spread on TTC plates and incubated at 30°C for 48 h. Finally, the bacterial colonies that formed were counted, and the bacterial content of the tobacco stems was calculated per unit gram.

### Determination of Physiological Parameters

Leaf samples were harvested at 0, 10, and 17 dpi. Subsequently, the activities peroxidase (POD), superoxide dismutase (SOD), phenylalanine ammonia-lyase (PAL), polyphenol oxidase (PPO), ascorbate peroxidase (APX), and catalase (CAT) were assessed. Also, the contents of malonaldehyde (MDA), chlorophyll, exopolysaccharides, and soluble proteins were determined. These assays were performed using specific assay kits (Nanjing Jiancheng Bioengineering Institute, Nanjing, China), following the manufacturer’s instructions. Mean values from five measurements were used for analyses. The results are presented as means ± SE of five biological and three technical replicates.

### Endogenous Hormone Measurement

To examine the levels of auxin (IAA), gibberellins (GA3), trans-zeatin riboside (ZR), abscisic acid (ABA), and salicylic acid (SA) in HR and MR genotypes, the leaves were harvested and immediately frozen in liquid nitrogen until further use. Sample extraction and hormone measurements were performed using enzyme-linked immunosorbent assays as previously described (Yu et al., 2016). The level of SA was determined using LC-MS analysis as previously described but with slight modifications (Cui et al., 2015). Briefly, plant materials (50 mg fresh weight) were frozen in liquid nitrogen, ground into fine powder, and extracted with 1 mL of methanol/water/formic acid (15:4:1, V/V/V). The combined extracts were evaporated to dryness under a nitrogen gas stream, reconstituted in 100 μL of 80% methanol (V/V), and filtered through a 0.22 μm filter for further LC-MS analysis.

Standard IAA, ZR, GA3, ABA, and SA (Sangon Biotech Co., Ltd., Shanghai, China) were used for calibration. The results are presented as means ± SE of five biological and three technical replicates.

### Transcriptomic Library Construction and Sequencing

Six samples (two genotypes × three biological replicates) were harvested at 0 dpi. Further, 24 samples (two genotypes × two treatments × two time points × three biological replicates) were harvested at 10 and 17 dpi. Finally, 30 samples were used for transcriptome sequencing. Total RNA was extracted from the stems of tobacco plants using TRIzol reagent (Invitrogen, Waltham, MA, United States). For RNA-seq library construction, 3 μg of total RNA was used and the library was sequenced on the Illumina HiSeq 2000 platform following the manufacturer’s protocol to yield ∼8 Gb of PE150 raw data. Sequencing data have been deposited in the NCBI Sequence Read Archive under accession number PRJNA762496.

### Bioinformatics Analysis of RNA-Seq Data

Clean reads were obtained by pre-processing raw reads to remove low-quality regions and adapter sequences. Clean reads were then mapped directly to the reference tobacco genome developed at the Yunnan Academy of Tobacco Agriculture Science (unpublished data) using HISAT2.0.5, and read counts of annotated genes were obtained. The expression level of each gene was measured in terms of FPKM (fragments per kilobase of transcript sequence per million base pairs sequenced). Using DESeq, transcripts with an adjusted *p*-value (padj) < 0.05 were identified as differentially expressed genes (DEGs) (Anders and Huber, 2010). Hierarchical clustering was conducted based on FPKM values. A Gene Ontology (GO) enrichment analysis of DEGs was conducted using the R package TopGO (Alexa et al., 2006) by an improved weighted scoring algorithm, using Fisher’s test to determine significance. A heatmap was generated using the R package Complex Heatmap 3.5.0 (Sun et al., 2016). The Kyoto Encyclopedia of Genes and Genomes (KEGG) database was used for functional enrichment analyses using Cluster Profiler 3.4.4. Values of *p*-value < 0.05 indicated significantly enriched pathways.

### Gene Expression Analysis

Total RNA was reverse-transcribed using the Prime Script™ RT Reagent Kit with gDNA Eraser (Perfect Real Time; Takara, Kusatsu, Japan) following the manufacturer’s protocol. Fourteen genes were randomly selected for validation by RT-qPCR using primers listed in Supplementary Table 1, as described previously (Pan et al., 2015). The relative gene expression levels were calculated using the comparative C_*T*_ method (Livak and Schmittgen, 2001). The ubiquitin-conjugating enzyme gene (*evm.TU.HIC_ASM_12.2258*) was used as the internal control for normalization of gene expression.

## Results

### Characterization of the Two Tobacco Cultivars in Response to *Ralstonia solanacearum* Infection

**FIGURE 1:**
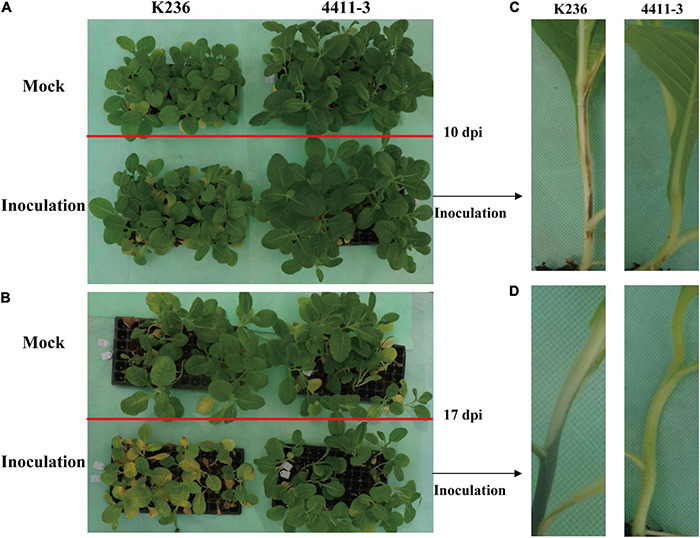
Disease symptoms in 4411-3 and K326 plants after *Ralstonia solanacearum* inoculation. **(A,B)** Tobacco seedlings in the pot at 10 and 17 days post inoculation (dpi) with *Ralstonia solanacearum*. **(C,D)** Disease symptoms in stems of 4411-3 and K326 plants at 10 and 17 dpi with *Ralstonia solanacearum*.

We initially conducted disease assays to confirm the response of the two *N*. *tabacum* L. cultivars (4411-3:HR and K326:MR) to *R. solanacearum* infection. A significant difference in the disease response was observed between the HR and MR genotypes at 17 dpi. All MR seedlings exhibited intense disease symptoms and ultimately died, whereas most HR seedlings showed no obvious disease symptoms (Figures 1A,B). Notably, MR stems exhibited black colors (wilt symptoms) at 10 and 17 dpi (Figures 1C,D). However, no similar symptom was detected in the HR stems (Figures 1C,D). These results confirm that the K326 variety is moderately resistant while the 4411-3 variety is highly resistant to *R. solanacearum* infection (Figure 1; Supplementary Figure 1 and Supplementary Table 2).

### Physiological Changes in the Two Tobacco Cultivars in Response to *Ralstonia solanacearum* Infection

ROS burst is associated with enhanced activities of anti-oxidative enzymes (Dumanović et al., 2020). To test this possibility in this study, we measured the activities of six anti-oxidative enzymes, including POD, CAT, SOD, PPO, PAL, and APX, which are typically activated to remove elevated ROS under oxidative stresses (Miller et al., 2010). Unexpectedly, our results showed that POD activity was significantly higher in the MR genotype than in the HR genotype at 10 and 17 dpi (Supplementary Figure 2A). Besides, there were no considerable differences in CAT and SOD levels between HR and MR genotype after *R. solanacearum* infection (Supplementary Figures 2B,C). PPO activity was significantly lower in MR than in HR (Supplementary Figure 2D), with no considerable differences in PAL and APX between the HR and MR genotypes after *R. solanacearum* infection (Supplementary Figures 2E,F). The unexpected results could be because the experiment was performed within a short duration, thus the exposure time was not sufficient to allow the desired physiological response.

At the three time points, the levels of chlorophyll differed significantly between the HR and MR genotypes (Supplementary Figure 3A) following to *R. solanacearum* infection. The loss of photosynthetic pigments may be due to the reduced number of living cells containing chloroplasts, as evidenced by necrotic lesions. Furthermore, the level of MDA increased sharply after *R. solanacearum* infection but was higher in the MR than in the HR genotype (Supplementary Figure 3B). Moreover, no considerable differences in soluble proteins and exopolysaccharides were noted between the two genotypes (Supplementary Figures 3C,D).

**FIGURE 2:**
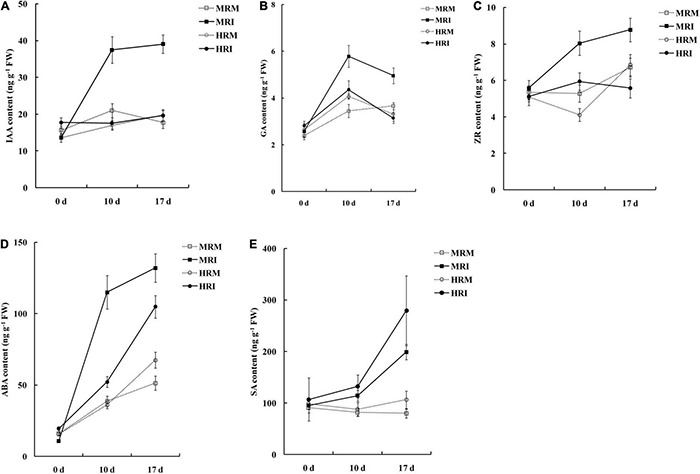
Hormone accumulation in the 4411-3 and K326 genotypes at 0, 10, and 17 days post-inoculation with *Ralstonia solanacearum*. **(A)** Auxin (IAA). **(B)** Gibberellins (GA3). **(C)** Trans-zeatin riboside (ZR). **(D)** Abscisic (ABA). **(E)** salicylic acid (SA). Five independent experimental replicates were analyzed for each sample and data are presented as means ± SE (standard error).

Plant hormones play pivotal signaling roles in host-*R. solanacearum* interactions (Zuluaga et al., 2015; French et al., 2018; Zhao et al., 2019). Therefore, we determined the levels of various hormones in the leaves of plants with the two genotypes. The contents of IAA, GA, ZR, and ABA in the MR genotype were significantly higher than those in the HR genotype after *R. solanacearum* infection (Figures 2A–D). Furthermore, SA levels in HR were significantly higher than those in the MR genotype in response to *R. solanacearum* infection (Figure 2E). These results indicate that resistance to *R. solanacearum* potentially results from the activation of the phytohormone-mediated pathway.

### Distinct Transcriptomes in 4411-3 and K326 Genotypes Under Mock Infection

We performed RNA-seq using RNA samples extracted from the stems of HR and MR genotypes at 0, 10, and17 dpi. In total, 1.80 billion clean reads (approximately 270.02 Gb of data) were obtained from 30 samples (Supplementary Table 3). Across all samples, the percentage of nucleotides with a quality score above 20 was over 97.86%, and the GC percentage ranged from 41.51% to 43.57% (Supplementary Table 3). After filtering and trimming, 84.74% to 95.32% of clean reads were uniquely mapped to the unpublished tobacco genome (Supplementary Table 4).

Furthermore, we performed RT-qPCR to compare the expression of 14 randomly selected genes with differential expression (DESeq2 padj ≤ 0.05 and fold change > 2) between the HR and MR genotypes. The results were consistent with those obtained by RNA-seq (Supplementary Table 5), supporting the reliability of the RNA-sequencing results.

**FIGURE 3:**
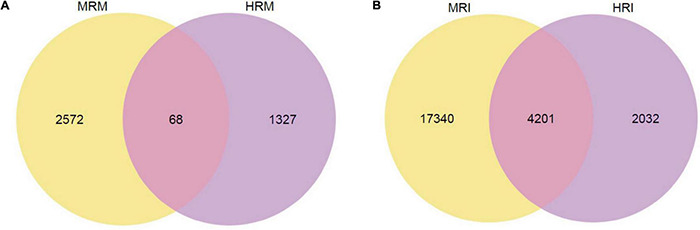
Venn diagram of total DEGs between the 4411-3 and K326 genotypes at 0, 10 and 17 days post-inoculation. **(A)** Venn diagram of total DEGs between the 4411-3 and K326 genotypes at 0, 10 and 17 days post-inoculation (dpi) under mock conditions (HRM vs. MRM). **(B)** Venn diagram of total DEGs between the 4411-3 and K326 genotypes at 0, 10 and 17 dpi after *Ralstonia solanacearum* inoculation (HRI vs. MRI).

Using DESeq2 padj ≤ 0.05 and fold change > 2 as thresholds, we found 3967 DEGs between the HR and MR genotypes at three time points. Among them, 1,395 and 2,640 had higher expression levels in the HR and MR genotypes, respectively (Figure 3A). These included 746 up-regulated genes in HR and 1981 up-regulated genes in MR at 0 dpi, 670 up-regulated genes in HR and 832 up-regulated genes in MR at 10 dpi, and 151 up-regulated genes in the HR genotype and 106 up-regulated genes in the MR genotype at 17 dpi (Supplementary Figures 4A–C and Supplementary Table 6). In addition, a hierarchical clustering algorithm revealed distinct expression profiles between the HR and MR genotypes (Supplementary Figure 5).

**FIGURE 4:**
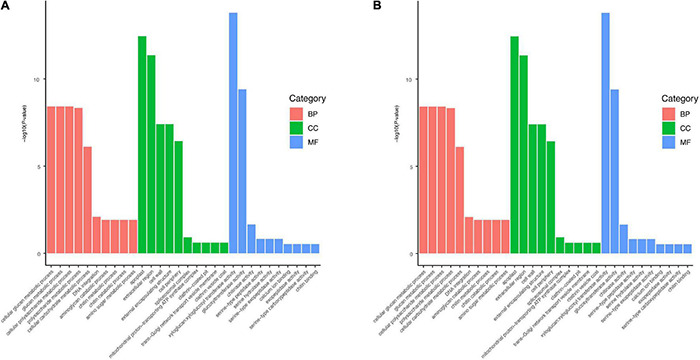
Significantly enriched GO terms for up-regulated genes in **(A)** 4411-3 genotype vs. **(B)** K326 genotype under mock conditions.

The DEGs between the HR and MR genotypes were further subjected to GO enrichment analysis. The up-regulated genes in the HR genotype were enriched in 26 biological processes (Supplementary Table 7). The terms cell wall macromolecule catabolic process, cell wall macromolecule metabolic process, and cell wall organization or biogenesis were over-represented (Figure 4A, Supplementary Table 7). The up-regulated genes in the MR genotype were enriched in 31 biological processes (Supplementary Table 8). Further, genes in the HR genotype were specifically enriched in the cellular component “cell wall” (Figure 4A, Supplementary Table 7). Within the molecular function category, three GO terms were significantly enriched: “xyloglucosyl transferase activity,” “glucosyltransferase activity,” and “chitinase activity” (Figure 4A, Supplementary Table 7). Genes in the MR genotype were significantly enriched in five GO terms in the cellular component category, including “DNA packaging complex” and “nucleosome” (Figure 4B, Supplementary Table 8). Nineteen molecular function GO terms were overrepresented in the MR genotype, including “microtubule motor activity” and “microtubule binding” (Figure 4B, Supplementary Table 8).

**FIGURE 5:**
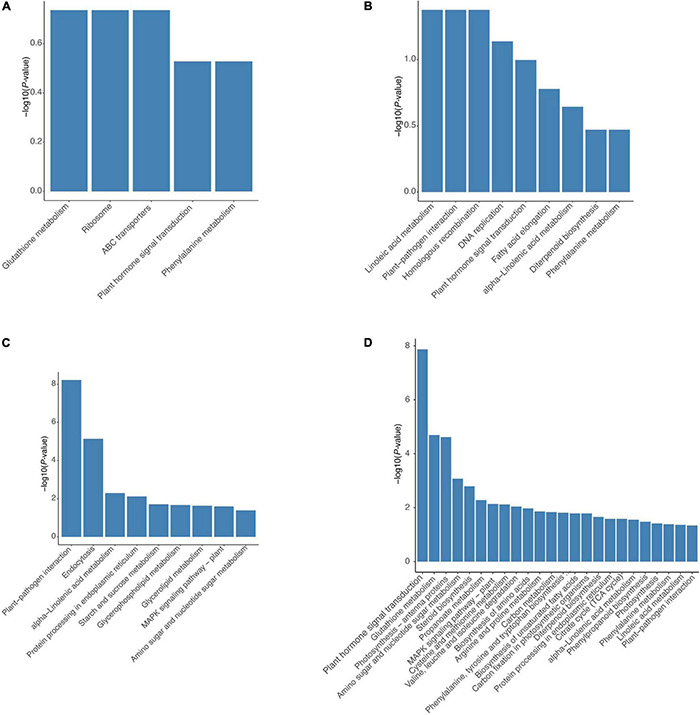
Significantly enriched KEGG pathways for DEGs in both 4411-3 and K326 genotype. Significantly enriched KEGG pathways for up-regulated genes in **(A)** the 4411-3 genotype vs. **(B)** the K326 genotype under mock conditions. Significantly enriched KEGG pathways for DEGs in **(C)** the 4411-3 genotype versus **(D)** the K326 genotype in response to *R. solanacearum* infection.

Kyoto Encyclopedia of Genes and Genomes pathway analysis was performed to further predict the functions of these DEGs. Up-regulated genes in the HR genotype were significantly enriched in five KEGG pathways, including glutathione metabolism, ribosome, ABC transporters, plant hormone signal transduction, and phenylalanine metabolism (Figure 5A). Up-regulated genes in the MR genotype were involved in nine KEGG pathways, including linoleic acid metabolism, plant-pathogen interaction, homologous recombination, DNA replication, plant hormone signal transduction, fatty acid elongation, alpha-linolenic acid metabolism, diterpenoid biosynthesis and phenylalanine metabolism (Figure 5B). Notably, three pathways, including glutathione metabolism, ribosome, and ABC transporters, were only enriched in the HR genotype (Figure 5). Genes related to plant hormone signal transduction and phenylalanine metabolism pathways were enriched in both tobacco genotypes (Figure 5).

### Identification of Differentially Expressed Genes Involved in the Response to *Ralstonia solanacearum* Infection

To examine transcriptome changes in both genotypes in response to *R. solanacearum* infection, we performed pairwise transcriptome comparisons between mock-treated and *R. solanacearum* inoculated plants at 10 and 17 dpi. In the HR genotype, 6,133 and 134 DEGs were identified in response to *R. solanacearum* infection at 10 and 17 dpi, respectively (Supplementary Figures 6A,B, Supplementary Table 9). In the MR genotype, 12,679 and 16,000 DEGs were identified at 10 and 17 dpi, respectively (Supplementary Figures 6C,D, Supplementary Table 10). In total, 6,233 and 21,541 non-redundant DEGs were identified in the HR and MR genotype, respectively, and 4,201 were common in both genotypes (Figure 3B).

Kyoto Encyclopedia of Genes and Genomes pathway analysis (*p* < 0.05) of 6,233 DEGs in the HR genotype under *R. solanacearum* infection revealed nine enriched pathways (Figure 5C). Meanwhile, 24 enriched pathways were detected among the 21,541 DEGs in the MR genotype following *R. solanacearum* infection (Figure 5D). Five pathways were enriched in both genotypes, including plant-pathogen interaction, alpha-linolenic acid metabolism, protein processing in endoplasmic reticulum, MAPK signaling pathway–plant, and amino sugar and nucleotide sugar metabolism (Figures 5C,D). Notably, four pathways were only enriched in the HR genotype, including endocytosis, starch and sucrose metabolism, glycerolipid metabolism, and glycerophospholipid metabolism (Figure 5C).

Moreover, we found that the expression patterns of 4,201 common DEGs were very similar in both genotypes at 10 dpi (Supplementary Figure 7A). KEGG pathway analysis of the 4,201 DEGs revealed eleven enriched pathways (Supplementary Figure 7B), including plant-pathogen interaction, endocytosis, protein processing in endoplasmic reticulum, starch and sucrose metabolism, phosphatidylinositol signaling system, explaining the common resistance of 4411-3 and K326 genotypes against *R. solanacearum* infection.

### Identification of Candidate Genes Related to *Ralstonia solanacearum* Resistance in Tobacco

According to the GO enrichment analysis, cell wall processing was over-represented in the up-regulated genes of the HR genotype but not in the MR genotype (Figure 4). The plant hormone signal transduction was selected according to KEGG pathway analysis and the physiological data obtained in this study (Figures 2, 5). According to the KEGG analysis, certain processes were specifically enriched in the HR genotype compared with MR genotype under mock and inoculation conditions (Figure 5). The enriched processed included glutathione metabolism, ABC transporters, glycerolipid metabolism, glycerophospholipid metabolism, and endocytosis. Therefore, the DEGs in these pathways were chosen as the candidate genes for further analysis.

**FIGURE 6:**
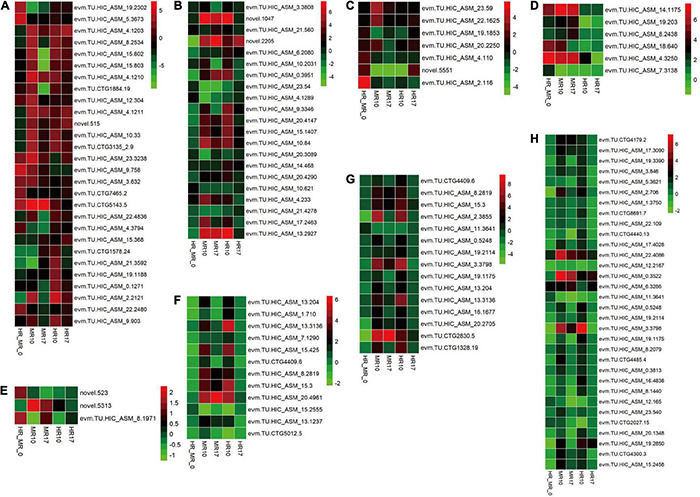
Comparison of resistance-related genes expressed in the highly resistant (4411-3, HR) and moderately resistant (K326, MR) genotype in response to *R. solanacearum* infection using a heatmap. Colors represent the log_2_ fold change values. HR_MR_0 = HRM_0 vs. MRM_0; MR10 = MRI_10 vs. MRM_10; MR17 = MRI_17 vs. MRM_17; HR10 (=HRI_10 vs. HRM_10; HR17 (=HRI_17 vs. HRM_17. **(A)** Cell wall-related genes. **(B)** Starch and sucrose metabolism-related genes. **(C)** Plant hormone signal transduction-related genes. **(D)** Glutathione metabolism-related genes. **(E)** ABC transporter-related genes. **(F)** Glycerolipid metabolism-related genes. **(G)** Glycerophospholipid metabolism-related genes. **(H)** Endocytosis-related genes.

Next, we evaluated candidate genes associated *R. solanacearum* resistance, including those related to the cell wall, starch and sucrose metabolism, plant hormone signal transduction, glutathione metabolism, ABC transporters, glycerolipid metabolism, glycerophospholipid metabolism, and endocytosis (Figure 6).

A total of 28 genes involved in cell wall processing were identified (Figure 6A, Supplementary Table 11), including 18 genes encoding xyloglucan endotransglycosylase/hydrolase (XTH), five genes encoding pectinesterase inhibitor, and five genes encoding pectinesterase. Additionally, the expression of 21 genes involved in starch and sucrose metabolism was altered in response to *R. solanacearum* infection (Figure 6B, Supplementary Table 11).

Seven genes involved in plant hormone signal transduction were identified (Figure 6C, Supplementary Table 11), including one JA-related gene, four ABA-related genes, and two auxin-related genes. Strikingly, the levels of the auxin-related genes *SAUR21* (small auxin up RNA21, novel.5551) and *SAUR24* (evm.TU.HIC_ASM_2.116) were 4.1- and 55.5-fold higher, respectively, in the HR than in the MR genotype (Figure 6C, Supplementary Table 11).

Six genes involved in glutathione metabolism were identified. These genes (e.g., genes encoding glutathione *S*-transferase) exhibited higher expression in the HR genotype than in the MR genotype (Figure 6D, Supplementary Table 11). Only three genes encoding ABC transporters were detected (Figure 6E, Supplementary Table 11). Furthermore, 12 genes involved in glycerolipid metabolism, 15 genes involved in glycerophospholipid metabolism, and 32 genes involved in endocytosis (Figures 6F–H, Supplementary Table 11) were identified.

## Discussion

### Common and Pathogen-Induced Transcriptomes Contribute to Tobacco Defense Against *Ralstonia solanacearum*

Few studies have characterized the interaction between tobacco and *R. solanacearum* at the molecular level (Nakano et al., 2013; Kiba et al., 2020; Zhuo et al., 2020; Wang Y. et al., 2021). In this study, a comparative transcriptomic analysis was performed to investigate the molecular mechanism underlying tobacco’s pathogen-induced responses to *R. solanacearum*. In the HR genotype, DEGs represent both common and HR-gene mediated responses, while DEGs in the MR genotype reflect the common defense responses and pathogen-dependent reprogramming in the plant.

A total of 3967 DEGs were found between the HR and MR genotype at 0 dpi, of which 1,395 and 2,640 were more expressed in the HR and MR genotypes, respectively (Figure 3A). These DEGs potentially contributed to the resistance and susceptibility in the two tobacco genotypes. Further, we found 6,133 DEGs in the HR genotype in response to *R. solanacearum* infection at 10 dpi, which was 45.8-fold more than the number detected at 17 dpi (Supplementary Figures 6A,B, Supplementary Table 9), suggesting that the HR genotype developed *R. solanacearum* resistance at an early stage. Interestingly, 6,233 and 21,541 non-redundant DEGs were identified in the HR and MR genotype in response to *R. solanacearum* infection at 10 and 17 dpi, respectively (Figure 3B). Overall, the number of DEGs was significantly higher in the MR than HR genotype, including 4,201 common DEGs, indicating that the two genotypes mounted different defense responses against *R. solanacearum*. DEGs involved in the response to *R. solanacearum* infection may contribute to genotypic differences in disease symptoms. Analysis of 4021 shared DEGs and 5 KEGG pathways revealed common sets of genes involved in the general defense response (Figures 3B, 5), indicating a complex and concerted response of the HR and MR genotypes to *R. solanacearum* infection. Notably, GO and KEGG analyses implicated seven specific pathways in the R genotype, including those associated with the cell wall, starch and sucrose metabolism, glutathione metabolism, ABC transporters, endocytosis, glycerolipid metabolism, and glycerophospholipid metabolism (Figures 4, 5). Collectively, these findings provide important basis for understanding the defense process.

### Cell Wall Biosynthesis May Be Linked to *Ralstonia solanacearum* Resistance in HR Tobacco Genotype

Cell wall is composed of hemicelluloses, cellulose microfibrils, and pectin. It provides a physical barrier to infection during pathogenic attack in plants (Molina et al., 2021). In this study, GO enrichment analysis indicated that many up-regulated genes in the HR genotype were involved in cell wall metabolism (Figures 4, 6A, Supplementary Table 11), including the cell wall macromolecule catabolic process, cell wall macromolecule metabolic process and cell wall organization or biogenesis. These findings are consistent with those of a previous study, which showed that genes associated with xyloglucan biosynthesis and cell wall organization were significantly enriched in response to *R. solanacearum* infection in pepper (Hwang et al., 2011). Also, cell wall-related genes showed genotype-specific expression differences between resistant and susceptible peanut (Chen et al., 2014).

In this study, some *XTH* genes were down-regulated in the MR genotype (Figure 6A, Supplementary Table 11). In addition, in terms of starch and sucrose metabolism, many genes involved in the biosynthesis of cell wall components exhibited different expression levels. For example, five of six enzymes (β-glucosidase) involved in cellulose hydrolysis were up-regulated in the HR genotype at 10 dpi (Figures 5, 6B, Supplementary Table 11), which was the opposite expression pattern reported in *Brassica oleracea* in response to *Plasmodiophora brassicae* infection (Zhang et al., 2016). Moreover, the DEGs related to the cell wall were also inhibited in the MR genotype. These results suggest that cell wall-related genes may confer different functions in the two genotypes in response to *R. solanacearum* infection.

### Plant Hormone Signal Transduction Pathways Participate in Tobacco-*Ralstonia solanacearum* Interactions and Initiate Defense Responses

Hormone crosstalk is crucial for plant defenses against pathogens (Robert-Seilaniantz et al., 2011). Auxin has also been implicated in the plant stress response but display complex plant-pathogen interactions patterns (Domingo et al., 2009; Kazan and Manners, 2009). In particular, root transcriptional analyses of *Arabidopsis*, tomato, and wild potato have demonstrated that hormone signaling pathways are altered upon contact with *R. solanacearum* (Zuluaga et al., 2015; French et al., 2018; Zhao et al., 2019), which may affect the root architecture and have direct or indirect effects on bacterial invasion.

Notably, SAUR may negatively regulate auxin biosynthesis and transport (Kant et al., 2009). In this study, the auxin-related genes *SAUR21* (*small auxin up RNA 21*, novel.5551) and *SAUR24* (evm.TU.HIC_ASM_2.116) were up-regulated 4.1- and 55.5-fold, respectively, in the HR genotype compared with the MR genotype. *SAUR21* and *SAUR24* were down-regulated 21.3- and 20.4-fold in MR genotype and R genotype at 10 dpi (Figure 6C, Supplementary Table 11). *SAUR24* was down-regulated 2.9- and 2.8-fold in MR and HR genotype at 17 dpi (Figure 6C, Supplementary Table 11). Consistently, we found that IAA accumulated in response to *R. solanacearum* infection, and this trend was stronger in the MR genotype than in the HR genotype (Figure 2A). These results indicate that an auxin signaling-mediated pathway may participate in the defense response of tobacco to *R. solanacearum*.

Transcriptomic analysis showed that *R. solanacearum* infection increased ABA-responsive gene expression in *Arabidopsis*, and several ABA receptor mutants with impaired ABA perception were more susceptible to *R. solanacearum* infection (Zhao et al., 2019). In this study, four ABA-related genes were highly expressed in the HR genotype in response to *R. solanacearum* infection. However, these genes showed expression changes in the MR genotype in response to *R. solanacearum* infection, in which two were up-regulated, while one was down-regulated (Figure 6C, Supplementary Table 11). These results show that ABA increases in response to *R. solanacearum* infection and this trend tends to be stronger in the MR genotype than in the HR genotype (Figure 2D).

Auxin was shown to be an important regulator of resistance to *R. solanacearum* infection in *Arabidopsis* (Zhao et al., 2019). Therefore, IAA and ABA may both participate in tobacco defense response against *R. solanacearum*. However, further studies are required to determine the precise role of these hormones in response to *R. solanacearum* infection in tobacco.

### Additional Candidate Genes Associated With Resistance to *Ralstonia solanacearum* in Tobacco

Glutathione *S*-transferases (GSTs) regulates cellular metabolism and are involved in various stress responses (Marrs, 1996). For example, GST gene cluster plays an important role in *Verticillium wilt* resistance in cotton (Li et al., 2019). In this study, all genes encoding GST for glutathione metabolism were up-regulated in the HR genotype compared with the MR genotype; five were up-regulated in the MR genotype at 10 dpi (Figure 6D, Supplementary Table 11). Therefore, differential expression of *GST* genes may be vital for BW resistance in tobacco. However, further experiments are needed to characterize their functions.

Some ABC transporters contribute to resistance against pathogens. The ABC transporter Lr34 confers resistance to multiple fungal pathogens in wheat (Krattinger et al., 2009). ABC transporters were highly expressed in barley upon inoculation with barley yellow dwarf virus (Wang et al., 2013). Consistent with the previous findings, ABC transporters (novel.523 and evm.TU.HIC_ASM_8.1971) were more expressed in the HR genotype than in the MR genotype and were strongly repressed in the MR genotype at 10 dpi (Figure 6E, Supplementary Table 11), suggesting that a resistance mechanism involving ABC transporters contributes to *R. solanacearum* resistance in tobacco.

Here, we found three additional pathways enriched explicitly in the HR genotype in response to *R. solanacearum* infection, including endocytosis, glycerolipid metabolism, and glycerophospholipid metabolism (Figures 6F–H, Supplementary Table 11), providing new insights into BW resistance in plants.

Studies have shown that “cell wall and plant hormone signal transduction pathways” regulates *R. solanacearum* resistance in *Arabidopsis* (Zhao et al., 2019), tomato (Wang and Xie, 2012; French et al., 2018), potato (Zuluaga et al., 2015), pepper (Hwang et al., 2011), and peanut (Chen et al., 2014). Glutathione metabolism and ABC transporters were also found to mediate resistance to other phytopathogens but not *R. solanacearum* infection (Krattinger et al., 2009; Wang et al., 2013; Supplementary Table 12). However, endocytosis, glycerolipid metabolism, and glycerophospholipid metabolism pathways have to been shown to regulate resistance against *R. solanacearum*, suggesting that these pathways may be specific to *R. solanacearum* resistance in tobacco. However, further studies are needed to verify these findings.

In conclusion, physiological indexes and transcriptomic analysis were performed to determine the mechanism underlying the response of tobacco to *R. solanacearum* infection. Numerous DEGs were detected in 4411-3 and K326 in response to *R. solanacearum* infection. The DEGs in the HR genotype were enriched in seven key pathways, including cell wall, starch and sucrose metabolism, glutathione metabolism, ABC transporters, endocytosis, glycerolipid metabolism, and glycerophospholipid metabolism. Notably, genes related to the cell wall, GST, and auxin synthesis potentially regulate complex resistance to *R. solanacearum* infection at the transcriptional level. Overall, these findings improve our understanding of the molecular mechanisms underlying the response of tobacco genotypes to *R. solanacearum* invasion and form the basis for identifying candidate genes involved in BW resistance in tobacco.

## Data Availability Statement

The original contributions presented in the study are publicly available. This data can be found here: National Center for Biotechnology Information (NCBI) BioProject database under accession number PRJNA762496.

## Author Contributions

ZeZ, XP, and JC designed the research. ZeZ, XP, JC, QY, WZ, TX, BC, MR, RG, ZaZ, ZM, and ZH performed the research. XP and ZeZ analyzed the data and wrote the manuscript. XP, ZeZ, and AY revised the manuscript. All authors approved the final manuscript.

## Conflict of Interest

The authors declare that the research was conducted in the absence of any commercial or financial relationships that could be construed as a potential conflict of interest.

## Publisher’s Note

All claims expressed in this article are solely those of the authors and do not necessarily represent those of their affiliated organizations, or those of the publisher, the editors and the reviewers. Any product that may be evaluated in this article, or claim that may be made by its manufacturer, is not guaranteed or endorsed by the publisher.

## Supplementary Material

Supplementary Figure 1 The bacterial content of stem tissue for RNA sequencing.

Supplementary Figure 2 Enzyme activities of POD **(A)**, CAT **(B)**, SOD **(C)**, PPO **(D)**, PAL **(E)**, and APX **(F)** in 4411-3 (HR) and K326 (MR) after *R. solanacearum* infection at 0, 10, and 17 days post-inoculation with *Ralstonia solanacearum*. Five independent experimental replicates were analyzed for each sample, and the data are presented as means ± SE (standard error). POD, peroxidase; CAT, catalase; SOD, superoxide dismutase; PPO, polyphenol oxidase; PAL, phenylalanine ammonia-lyase; APX, ascorbate peroxidase.

Supplementary Figure 3 Contents of chlorophyll **(A)**, MDA **(B)**, soluble protein **(C)**, and exopolysaccharides **(D)** at 0, 10, and 17 days post-inoculation with *Ralstonia solanacearum*. Five independent experimental replicates were analyzed for each sample, and the data are presented as means ± SE (standard error). MDA, malondialdehyde.

Supplementary Figure 4 Volcano plot of differentially expressed genes (DEGs) between 4411-3 (HR) genotype and K326 (MR) genotypes under mock conditions. **(A)** Volcano plot of differentially expressed genes (DEGs)between 4411-3 (HR) genotype and K326 (MR) genotypes at day 0 under mock conditions (HRM_0 vs. MRM_0). **(B)** Volcano plot of differentially expressed genes (DEGs)between the 4411-3 (HR) and K326 (MR) genotypes at day 10 under mock conditions(HRM_10 vs. MRM_10). **(C)** Volcano plot of differentially expressed genes (DEGs)between the 4411-3 (HR) and K326 (MR) genotypes at day 17 under mock conditions(HRM_17 vs. MRM_17).

Supplementary Figure 5 Transcriptome profile analysis of both 4411-3 and K326 genotypes under mock conditions at 0, 10, and 17 days. Blue indicates low expression, white indicates intermediate expression, and red indicates high expression (FPKM > 1).

Supplementary Figure 6 Volcano plot of differentially expressed genes (DEGs) in 4411-3 (HR) and K326 (MR) after *Ralstonia solanacearum* infection. **(A)** Volcano plot of differentially expressed genes (DEGs) in 4411-3 (HR) at 10 days post-inoculation with *Ralstonia solanacearum*. (HRI_10 vs. HRM_10). **(B)** Volcano plot of differentially expressed genes (DEGs) in 4411-3 (HR) at 17 days post-inoculation with *Ralstonia solanacearum*. (HRI_17 vs. HRM_17). **(C)** Volcano plot of differentially expressed genes (DEGs) in K326 (MR) at 10 days post-inoculation with *Ralstonia solanacearum*. (MRI_10 vs. MRM_10). **(D)** Volcano plot of differentially expressed genes (DEGs) in K326 (MR) at 17 days post-inoculation with *Ralstonia solanacearum*. (MRI_17 vs. MRM_17).

Supplementary Figure 7 Transcriptome profile analysis and KEGG pathways of 4201 common genes in 4411-3 and K326 genotypes after *Ralstonia solanacearum* infection. **(A)** Transcriptome profile analysis of 4201 common genes in 4411-3 and K326 genotypes after *Ralstonia solanacearum* infection at 0, 10, and 17 days. Blue indicates low expression, white indicates intermediate expression, and red indicates high expression (FPKM > 1). **(B)** Significantly enriched KEGG pathways for 4201 common DEGs in the 4411-3 and K326 genotype in response to *Ralstonia solanacearum* infection.

Supplementary Table 1 Sequences of primers used for RT-qPCR.

Supplementary Table 2 Evaluation of disease resistance for experimental materials.

Supplementary Table 3 Quality parameters for RNA-seq raw data.

Supplementary Table 4 List of mapping results.

Supplementary Table 5 List of genes validated by qRT-PCR to verify the reliability of RNA-seq data.

Supplementary Table 6 List of differentially expressed genes (DEGs) in 4411-3 (HR) vs. K326 (MR) under mock conditions at 0, 10, and 17 days. HR_MR_0 = HRM_0 vs. MRM_0; HR_MR_10 = HRM_10 vs. MRM_10; HR_MR_17 = HRM_17 vs. MRM_17.

Supplementary Table 7 List of significantly enriched GO terms for up-regulated genes in 4411-3 (HR) vs. K326 (MR) under mock conditions.

Supplementary Table 8 List of significantly enriched GO terms for up-regulated genes in K326 (MR) vs. 4411-3 (HR) under mock conditions.

Supplementary Table 9 List of differentially expressed genes (DEGs) in 4411-3 (HR) at 10 and 17 days post inoculation with *Ralstonia solanacearum*. HR10 = HRI_10 vs. HRM_10; HR17 = HRI_17 vs. HRM_17.

Supplementary Table 10 List of differentially expressed genes (DEGs) in K326 (MR) at 10 and 17 days post inoculation with *Ralstonia solanacearum*. MR10 = MRI_10 vs. MRM_10; MR17 = MRI_17 vs. MRM_17.

Supplementary Table 11 List of resistance-related genes expressed in 4411-3 (HR) and K326 (MR) in response to *Ralstonia solanacearum* infection.

Supplementary Table 12 Significantly enriched GO terms after *Ralstonia solanacearum* infection in *Arabidopsis*, tomato, potato, pepper, and peanut.
