# Supplementary material for: Comparative Transcriptome Profiling Reveals Defense-Related Genes Against Ralstonia solanacearum Infection in Tobacco
Source: Front Plant Sci. 2021 Dec 14;12:767882. doi: 10.3389/fpls.2021.767882 (PMC8712766; doi:10.3389/fpls.2021.767882)

**Supplementary Figure S1** The bacterial content of stem tissue for RNA sequencing.

**Supplementary Figure S2** Enzyme activities of POD (**A**), CAT (**B**), SOD (**C**), PPO (**D**), PAL (**E**), and APX (**F**) in 4411-3 (HR) and K326 (MR) after *R. solanacearum* infection at 0, 10, and 17 days post-inoculation with *Ralstonia solanacearum*.

**Supplementary Figure S3** Contents of chlorophyll (A), MDA (B), soluble protein (C), and exopolysaccharides (D) at 0, 10, and 17 days post-inoculation with *Ralstonia solanacearum*.

**Supplementary Figure S4** Volcano plot of differentially expressed genes (DEGs) between 4411-3 (HR) genotype and K326 (MR) genotypes under mock conditions.

R=HR (4411-3), S=MR (K326)

**Supplementary Figure S5** Transcriptome profile analysis of both 4411-3 and K326 genotypes under mock conditions at 0, 10, and 17 days.

R=HR (4411-3), S=MR (K326)

**Supplementary Figure S6** Volcano plot of differentially expressed genes (DEGs) in 4411-3 (HR) and K326 (MR) after *Ralstonia solanacearum* infection.

R=HR (4411-3), S=MR (K326)

**Supplementary Figure S7** Transcriptome profile analysis and KEGG pathways of 4201 common genes in 4411-3 and K326 genotypes after *Ralstonia solanacearum* infection.

R=HR (4411-3), S=MR (K326)

**A**


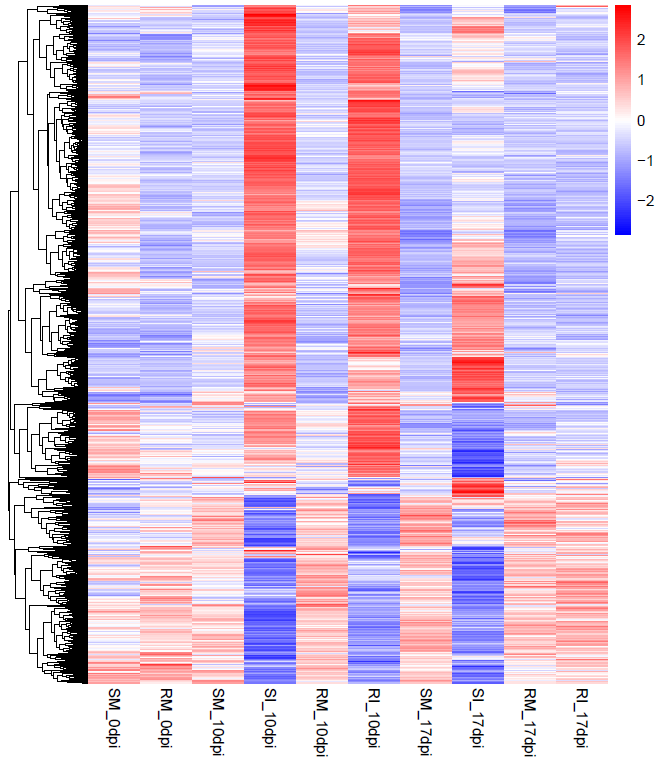


**B**


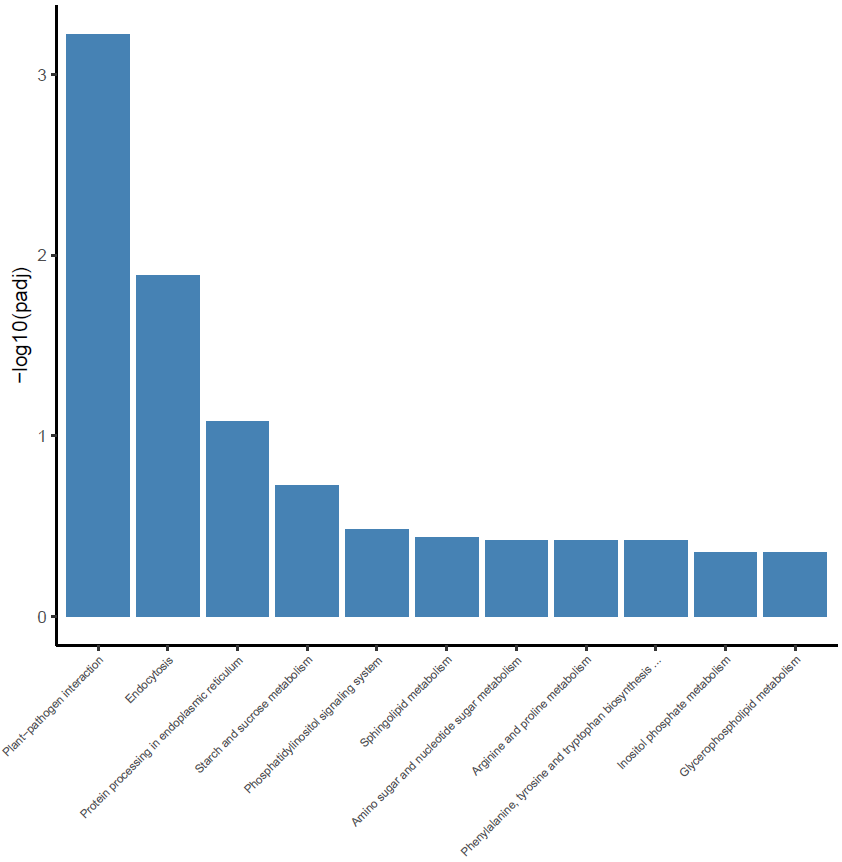

Supplement: Supplementary Figure 1 — The bacterial content of stem tissue for RNA sequencing. [file Data_Sheet_1.doc]
