# Supplementary material for: Comparative Transcriptome Profiling Reveals Defense-Related Genes Against Ralstonia solanacearum Infection in Tobacco
Source: Front Plant Sci. 2021 Dec 14;12:767882. doi: 10.3389/fpls.2021.767882 (PMC8712766; doi:10.3389/fpls.2021.767882)
Supplement: Supplementary Table 2 — Evaluation of disease resistance for experimental materials. [file Table_2.doc]

**Supplementary Table S2** Evaluation of disease resistance for experimental materials.

|  | 10 d | | 17 d | |
| --- | --- | --- | --- | --- |
|  | RDP (%) | DI | RDP (%) | DI |
| K326 | 8.51 | 1.43 | 52.81 | 22.68 |
| 4411-3 | 0 | 0 | 2.08 | 0.23 |
