# Supplementary material for: Comparative Transcriptome Profiling Reveals Defense-Related Genes Against Ralstonia solanacearum Infection in Tobacco
Source: Front Plant Sci. 2021 Dec 14;12:767882. doi: 10.3389/fpls.2021.767882 (PMC8712766; doi:10.3389/fpls.2021.767882)
Supplement: Supplementary Table 3 — Quality parameters for RNA-seq raw data. [file Table_3.doc]

**Supplementary Table S3 Quality parameters for RNA-seq raw data.**

| sample | raw_reads | clean_reads | clean_bases | error_rate | Q20 | Q30 | GC_pct |
| --- | --- | --- | --- | --- | --- | --- | --- |
| MRM_0dpi_1 | 62344488 | 60068606 | 9.01G | 0.02 | 98.11 | 94.25 | 42.74 |
| MRM_0dpi_2 | 59749300 | 57693550 | 8.65G | 0.03 | 98.03 | 94.14 | 42.31 |
| MRM_0dpi_3 | 64421000 | 61373000 | 9.21G | 0.02 | 98.06 | 94.11 | 42.71 |
| HRM_0dpi_1 | 61089222 | 59127572 | 8.87G | 0.03 | 97.98 | 93.96 | 42.41 |
| HRM_0dpi_2 | 61196318 | 59397134 | 8.91G | 0.03 | 97.87 | 93.7 | 43.17 |
| HRM_0dpi_3 | 63790710 | 62160494 | 9.32G | 0.03 | 97.86 | 93.8 | 43.1 |
| MRM_10dpi_1 | 62693548 | 61132618 | 9.17G | 0.03 | 98.04 | 94.04 | 42.44 |
| MRM_10dpi_2 | 60935396 | 59458354 | 8.92G | 0.03 | 98.04 | 94.1 | 42.56 |
| MRM_10dpi_3 | 61558434 | 60074442 | 9.01G | 0.03 | 98.07 | 94.08 | 42.7 |
| HRM_10dpi_1 | 60454724 | 58656212 | 8.8G | 0.03 | 98.01 | 94.09 | 42.48 |
| HRM_10dpi_2 | 63456468 | 61785660 | 9.27G | 0.03 | 98.01 | 93.97 | 43.57 |
| HRM_10dpi_3 | 62840952 | 61107868 | 9.17G | 0.03 | 97.99 | 93.96 | 41.81 |
| MRI_10dpi_1 | 59664510 | 57964870 | 8.69G | 0.03 | 97.98 | 93.93 | 42.52 |
| MRI_10dpi_2 | 61285284 | 58822358 | 8.82G | 0.03 | 97.99 | 93.95 | 41.73 |
| MRI_10dpi_3 | 61793784 | 59834950 | 8.98G | 0.03 | 98 | 94.06 | 42.44 |
| HRI_10dpi_1 | 65681164 | 64027196 | 9.6G | 0.03 | 98.06 | 94.08 | 42.49 |
| HRI_10dpi_2 | 59197388 | 57663638 | 8.65G | 0.02 | 98.12 | 94.15 | 42.46 |
| HRI_10dpi_3 | 63198852 | 61039112 | 9.16G | 0.03 | 97.96 | 93.98 | 41.96 |
| MRM_17dpi_1 | 56217728 | 54792580 | 8.22G | 0.03 | 97.94 | 93.87 | 42.4 |
| MRM_17dpi_2 | 64105564 | 61787874 | 9.27G | 0.03 | 97.94 | 93.76 | 42.16 |
| MRM_17dpi_3 | 64847618 | 63099458 | 9.46G | 0.03 | 97.98 | 93.93 | 42.16 |
| HRM_17dpi_1 | 64154304 | 62041082 | 9.31G | 0.03 | 97.97 | 93.88 | 41.82 |
| HRM_17dpi_2 | 63122722 | 60507200 | 9.08G | 0.02 | 98.05 | 94.15 | 43.45 |
| HRM_17dpi_3 | 61692688 | 59256426 | 8.89G | 0.02 | 98.08 | 94.12 | 42.06 |
| MRI_17dpi_1 | 61743598 | 59103932 | 8.87G | 0.02 | 98.17 | 94.33 | 41.69 |
| MRI_17dpi_2 | 61452936 | 58673444 | 8.8G | 0.02 | 98.05 | 94.11 | 42.03 |
| MRI_17dpi_3 | 63445776 | 60488184 | 9.07G | 0.02 | 98.16 | 94.31 | 41.51 |
| HRI_17dpi_1 | 62074118 | 59662870 | 8.95G | 0.03 | 97.89 | 93.77 | 42.77 |
| HRI_17dpi_2 | 62016250 | 59973188 | 9.0G | 0.03 | 97.92 | 93.81 | 42.5 |
| HRI_17dpi_3 | 61165490 | 59252420 | 8.89G | 0.03 | 97.91 | 93.75 | 42.48 |
