# Supplementary material for: Comparative Transcriptome Profiling Reveals Defense-Related Genes Against Ralstonia solanacearum Infection in Tobacco
Source: Front Plant Sci. 2021 Dec 14;12:767882. doi: 10.3389/fpls.2021.767882 (PMC8712766; doi:10.3389/fpls.2021.767882)
Supplement: Supplementary Table 4 — List of mapping results. [file Table_4.doc]

**Supplementary Table S4 List of mapping results.**

| sample | total_reads | total_map | unique_map | multi_map | positive_map | negative_map |
| --- | --- | --- | --- | --- | --- | --- |
| MRM_0dpi_1 | 60068606 | 56826887(94.6%) | 55284007(92.03%) | 1542880(2.57%) | 27627481(45.99%) | 27656526(46.04%) |
| MRM_0dpi_2 | 57693550 | 56239164(97.48%) | 54684746(94.78%) | 1554418(2.69%) | 27313292(47.34%) | 27371454(47.44%) |
| MRM_0dpi_3 | 61373000 | 57068930(92.99%) | 55364514(90.21%) | 1704416(2.78%) | 27661816(45.07%) | 27702698(45.14%) |
| HRM_0dpi_1 | 59127572 | 56530390(95.61%) | 55183076(93.33%) | 1347314(2.28%) | 27571258(46.63%) | 27611818(46.7%) |
| HRM_0dpi_2 | 59397134 | 54011294(90.93%) | 52442861(88.29%) | 1568433(2.64%) | 26200090(44.11%) | 26242771(44.18%) |
| HRM_0dpi_3 | 62160494 | 58202471(93.63%) | 56690144(91.2%) | 1512327(2.43%) | 28314507(45.55%) | 28375637(45.65%) |
| MRM_10dpi_1 | 61132618 | 59492153(97.32%) | 57539230(94.12%) | 1952923(3.19%) | 28748385(47.03%) | 28790845(47.1%) |
| MRM_10dpi_2 | 59458354 | 57727286(97.09%) | 56215257(94.55%) | 1512029(2.54%) | 28090195(47.24%) | 28125062(47.3%) |
| MRM_10dpi_3 | 60074442 | 58920818(98.08%) | 57264999(95.32%) | 1655819(2.76%) | 28616885(47.64%) | 28648114(47.69%) |
| HRM_10dpi_1 | 58656212 | 55935870(95.36%) | 54492559(92.9%) | 1443311(2.46%) | 27224104(46.41%) | 27268455(46.49%) |
| HRM_10dpi_2 | 61785660 | 54526677(88.25%) | 53022864(85.82%) | 1503813(2.43%) | 26490386(42.87%) | 26532478(42.94%) |
| HRM_10dpi_3 | 61107868 | 58877115(96.35%) | 57498927(94.09%) | 1378188(2.26%) | 28726821(47.01%) | 28772106(47.08%) |
| MRI_10dpi_1 | 57964870 | 56650876(97.73%) | 54097670(93.33%) | 2553206(4.4%) | 27032828(46.64%) | 27064842(46.69%) |
| MRI_10dpi_2 | 58822358 | 56815799(96.59%) | 54975825(93.46%) | 1839974(3.13%) | 27460824(46.68%) | 27515001(46.78%) |
| MRI_10dpi_3 | 59834950 | 56754572(94.85%) | 54302639(90.75%) | 2451933(4.1%) | 27138189(45.36%) | 27164450(45.4%) |
| HRI_10dpi_1 | 64027196 | 62572743(97.73%) | 60729194(94.85%) | 1843549(2.88%) | 30347400(47.4%) | 30381794(47.45%) |
| HRI_10dpi_2 | 57663638 | 56526977(98.03%) | 54964344(95.32%) | 1562633(2.71%) | 27469713(47.64%) | 27494631(47.68%) |
| HRI_10dpi_3 | 61039112 | 58213544(95.37%) | 56632551(92.78%) | 1580993(2.59%) | 28287534(46.34%) | 28345017(46.44%) |
| MRM_17dpi_1 | 54792580 | 53401440(97.46%) | 51884357(94.69%) | 1517083(2.77%) | 25920694(47.31%) | 25963663(47.39%) |
| MRM_17dpi_2 | 61787874 | 60540135(97.98%) | 58891943(95.31%) | 1648192(2.67%) | 29428020(47.63%) | 29463923(47.69%) |
| MRM_17dpi_3 | 63099458 | 61429405(97.35%) | 59588330(94.44%) | 1841075(2.92%) | 29766434(47.17%) | 29821896(47.26%) |
| HRM_17dpi_1 | 62041082 | 56740502(91.46%) | 55154568(88.9%) | 1585934(2.56%) | 27557889(44.42%) | 27596679(44.48%) |
| HRM_17dpi_2 | 60507200 | 52698341(87.09%) | 51275450(84.74%) | 1422891(2.35%) | 25614434(42.33%) | 25661016(42.41%) |
| HRM_17dpi_3 | 59256426 | 58091014(98.03%) | 56251469(94.93%) | 1839545(3.1%) | 28102942(47.43%) | 28148527(47.5%) |
| MRI_17dpi_1 | 59103932 | 57750410(97.71%) | 54719149(92.58%) | 3031261(5.13%) | 27343466(46.26%) | 27375683(46.32%) |
| MRI_17dpi_2 | 58673444 | 57306418(97.67%) | 54680932(93.2%) | 2625486(4.47%) | 27318385(46.56%) | 27362547(46.64%) |
| MRI_17dpi_3 | 60488184 | 58895179(97.37%) | 55097534(91.09%) | 3797645(6.28%) | 27534953(45.52%) | 27562581(45.57%) |
| HRI_17dpi_1 | 59662870 | 57317108(96.07%) | 55690673(93.34%) | 1626435(2.73%) | 27820162(46.63%) | 27870511(46.71%) |
| HRI_17dpi_2 | 59973188 | 55408978(92.39%) | 54049391(90.12%) | 1359587(2.27%) | 27005030(45.03%) | 27044361(45.09%) |
| HRI_17dpi_3 | 59252420 | 57168724(96.48%) | 55773054(94.13%) | 1395670(2.36%) | 27865448(47.03%) | 27907606(47.1%) |
